# Adverse effect of *Tamarindus* *indica* and tamoxifen combination on redox balance and genotoxicity of breast cancer cell

**DOI:** 10.1186/s43141-023-00564-z

**Published:** 2023-11-21

**Authors:** R. A. Guneidy, E. R. Zaki, G. S. A. Abdel Karim, N. S. Saleh, A. Shokeer

**Affiliations:** Department of Molecular Biology, Biotechnology Research Institute, National Research Centre, Cairo, Egypt

**Keywords:** Breast cancer, Combination treatment, Glutathione-related enzymes, Genotoxicity, Oxidative stress, Plant cytotoxicity, Tamoxifen

## Abstract

**Background:**

Breast cancer is the most significant threat to women worldwide. Most chemotherapeutic drugs cause cancer cell death and apoptosis by inducing oxidative stress and producing reactive oxygen species (ROS). Cancer cells have a higher rate of metabolic activity than normal cells and thus produce more ROS. Glutathione and its related enzymes are the most significant antioxidant defense mechanisms that protect cells from oxidative and chemotherapeutic impacts. The anticancer actions of phenolic compounds were greatly confirmed. Using phenolic compounds as drugs in combination with chemotherapy may improve health, improve treatment outcomes, and reduce dose and damage. The goal of the study was to treat breast cancer cell lines (MCF-7) with *Tamarindus indica* extract individually and in combination with the anticancer drug tamoxifen (TAM) to improve therapeutic efficacy.

**Results:**

After 48 h of incubation at IC_25_ concentrations of *T. indica* extract (47.3 g/mL), tamoxifen (0.8 g/mL), and their co-treatments, the biochemical and genotoxic effects on MCF-7 cell lines were investigated. In MCF7 cell lines, *T. indica* extract increased reduced glutathione levels as well as glutathione transferase, glutathione peroxidase, and glutathione reductase activities. The same was true for oxidative state indicators, where higher levels of catalase and lactate dehydrogenase activity were associated with higher levels of malondialdehyde. *T. indica* has almost no effect on the DNA damage parameters. All of these variations can produce alterations in cancer cell genotoxicity and apoptotic pathways, explaining the restoration of DNA moment to normal levels and enhanced survival.

**Conclusion:**

Cytotoxic and genotoxic effect of treatment with *T. indica* extract could be attributed to the dynamic interaction of glutathione cycle and antioxidant enzymes to combat oxidative stress, which can be considered as a positive therapeutic effect. On the other hand, the negative response of tamoxifen efficacy when co-treated with *T. indica* reversed tamoxifen’s genotoxicity and enhanced survival.

## Background

Cancer is a growing health problem in both developing and developed countries. It is one of the most common diseases and causes of death, causing the death of nearly 10 million people annually [1, 2]. Breast cancer is the most common type of cancer in women and the major reason for death among women. It accounts for 33% of female cancer cases in Egypt, with over 22,000 new cases identified each year. This is expected to rise significantly in the coming years as the population grows, the population pyramid shifts, and people embrace unhealthy lifestyles. Despite significant progress in survival rates in many developed countries, the 5-year survival rate in Egypt is still between 28 and 68% [3].

Cancer drugs generally either inhibit or kill cancer cell development. There are various types of anticancer drugs, including alkylating agents, antimetabolic, cytotoxic drugs, plant derivatives, and protein kinase inhibitors [4]. Tamoxifen (TAM), an anti-estrogen agent, is a drug commonly used in the treatment of breast cancer due to its inhibitory effects on estrogen receptor transcription activity. Adjunctive therapy with tamoxifen has previously been shown to be effective against early-stage breast cancer and with prolonged survival. However, some breast cancer patients develop resistance to tamoxifen during long-term treatment with TAM [5].

Multidrug resistance (MDR) is considered the most serious problem in cancer cells. It affects the therapeutic efficacy of anticancer drugs and is a serious barrier to cancer treatment. The development of redox imbalances and oxidative stress, increased levels of detoxifying enzymes, changes in drug efflux, changes in drug target enzymes, improved DNA repair, and deactivation of apoptotic pathways are the most common MDR resistance mechanisms [6–9]. Most chemotherapeutic drugs induce cancer cell death and apoptosis through the induction of oxidative stress and the generation of reactive oxygen species (ROS). Oxidative stress causes an unstable oxidant-antioxidant balance in favor of oxidizing substances, leading to increased production of free radicals, particularly ROS (H_2_O_2_, O2^•−^, OH^•^, etc.), thus damage to biomolecules. Cancer cells have a higher rate of metabolic activity compared to normal cells; they exhibit higher rates of oxidative stress and ROS production. It is believed that in cancer cells, ROS may play a dual role by eliciting both pro-apoptotic and pro-survival effects. Unregulated redox systems and altered intracellular ROS levels have been defined as underlying mechanisms mediating MDR, which regulates key cellular pathways involved in drug resistance and pro-survival regulating pathways. Therefore, redox-modifying strategies are required to overcome this resistance and to improve cancer drug sensitization [7, 10]. The most important antioxidant mechanism that protects cells from free radical, radiation, and chemotherapy attacks is glutathione (GSH) and its related enzymes. Cancer cell GSH content is directly related to the overall regulation of redox status and drug response [11].

In recent decades, the limited efficacy of some drugs and the severity of side effects have drawn attention to naturally derived compounds [12, 13]. Combined chemotherapy with certain natural compounds may improve health, reduce the chance of recurrence, improve chemotherapy outcomes, and reduce dose and damage [4]. The anticancer effects of different polyphenols were widely improved as greatly beneficial and interesting agents in attack cancer cells by a variety of ways [9]. They were identified as redox-active molecules with relatively low toxicity. They exhibit a wide range of benefits including anti-inflammatory, antimutagenic, and anticarcinogenic actions [7, 13–15].

Combination therapy with tamoxifen and antioxidants can enhance the anticancer efficacy of tamoxifen as the administration of tamoxifen in combination with antioxidants such as riboflavin and niacin restores antioxidant activity accompanied by enhanced antitumor activity. However, antioxidants can reduce the effectiveness of anticancer treatments [16].

*Tamarindus indica* (*T. indica*) is a well-known medicinal plant which is a perennial tropical plant that is widely taken as a traditional medicinal plant all over the world. The chemistry of this plant falls into several classes, including phenolic compounds and flavonoids [17]. Previously, *T. indica* extract was shown to have antioxidant activity and cytotoxic effect on breast cancer cell lines (MCF-7), where it inhibited the activity of these cells by 72%. However, the combination therapy of *T. indica* extract with the drug tamoxifen yielded unexpected results. Both the plant extract and the cytotoxic inhibitory effect of tamoxifen were eliminated [18].

The aim of this study was to evaluate the effect of *T. indica* extract on one of the most important antioxidant defense mechanisms, GSH, and its related enzymes responsible for cell defense against oxidative stress and the effects of chemotherapy. Additionally, the study investigated the effect of combination therapy using tamoxifen with the plant extract to assess its impact on increasing tamoxifen efficacy, reducing drug dose, and addressing drug resistance, which is a significant contributor to cancer treatment failure. GSH was added to the extract and TAM combination therapy, assuming that it would reduce the negative effects of this combination. GSH is available as a dietary supplement to improve the efficiency of anti-toxin systems.

## Materials and methods

### Chemicals

Dichloromethane, ethyl acetate, ethanol, and *n*-butanol, bovine serum albumin fraction ΙV (BSA), reduced glutathione (GSH), oxidized glutathione (GSSG), and 1-chloro-2, 4-dinitrobenzene (CDNB) were purchased from Merck Company. Nicotinamide adenine dinucleotide phosphate reduced form (NADPH) and tamoxifen were purchased from Sigma Chemical Company (St. Louis, MO, USA). All other chemicals were of the highest purity commercially available.

### Preparation of plant extracts

Dry seeds of *T. indica*, a well-known plant in the local markets in Egypt with traditional uses, were purchased and identified. *T. indica* seeds (250 g) were washed with water, dried at room temperature for 6 days and ground into fine powder using a domestic blender, and mixed with solvents (1:5 w/v) of increasing polarity: dichloromethane, ethyl acetate, and *n*-butanol. The mixture was allowed to stand at room temperature for 12 h in the dark, with occasional agitation. The partitioned fractions were centrifuged at 1000 × *g* for 10 min, filtered through Whatman No. 1 filter paper, and evaporated to dryness. The obtained dry weights (DW) of the three fractions were weighted and saved at − 4 °C for further analysis.

### Cell line authentication, culture, and treatment

A human breast cancer cell line, human Caucasian breast cancer, MCF-7, was established. All the following procedures were done in a sterile area using a laminar flow cabinet biosafety class II level (Baker, SG403INT, Sanford, ME, USA). Cells were suspended in Dulbecco’s Modified Eagle’s Medium–high glucose (DMEM medium) HCT116, 1% antibiotic–antimycotic mixture (10,000 U/ml potassium penicillin, 10,000 µg/ml streptomycin sulfate and 25 µg/ml amphotericin B), and 1% L-glutamine at 37 °C under 5% CO_2_. Cells were batch cultured for 10 days and then seeded at concentration of 10 × 10^3^ cells/well in fresh complete growth medium in 96-well microtiter plastic plates at 37 °C for 24 h under 5% CO_2_ using a water-jacketed carbon dioxide incubator (Sheldon, TC2323, Cornelius, OR, USA) [19]. Media was aspirated, fresh medium (without serum) was added, and cells were incubated for 48 h either alone (negative control) or with different IC_25_ concentrations of *T. indica* extract (47.3 g/mL) TAM (0.8 µg/mL) and their co-treatments in the absence and presence of GSH (100 µM).

### Preparation of cell lysate

After incubation, the medium was removed. Cells were scraped. The cells were washed twice with cold phosphate buffer saline. Cells were lysed in 0.1-M potassium phosphate buffer, pH 8.0 containing 5.0-mM EDTA and 5.0-mM β-mercaptoethanol. The lysate was sonicated for 30 s three times, centrifuged at 2000 rpm, and preserved at − 20 °C for further analyses.

### Biochemical analyses

#### Glutathione transferase activity

Glutathione transferase (GSTs; EC 2.5.1.18) activity was determined according to the method [20] by measuring the increase in the concentration of the conjugation product of GSH and CDNB at 340 nm over 3 min at 30 °C. One unit of GST activity is defined as the formation of 1 µmole product per min at 30 °C.

#### Glutathione peroxidase activity

The activity of glutathione peroxidase (GPx; EC 1.11.1.9) was determined according to the method described by [21]. The assay reaction mixture contained in1-mL volume, 50-mM potassium phosphate buffer, pH 7.0, 0.005-M EDTA, 0.075-mM H_2_O_2_, 5.0-mM GSH, 0.28-mM NADPH, 1-IU GR, and a suitable crude enzyme homogenate volume. One unit is equivalent to the oxidation of 1 µmole of NADPH in 1 min, at 30 °C. The extinction coefficient of NADPH was taken to be 6.22 mM^−1^ cm^−1^.

#### Glutathione reductase activity

The activity of glutathione reductase (GR; EC 1.8.1.7) was determined spectrophotometrically at 30 °C following the decrease in absorbance at 340 nm according to the method described by [22]. The assay reaction mixture contained in a total volume of 1 mL, 50-mM potassium phosphate buffer, pH 7.0, 1-mM EDTA, 0.1-mM NADPH, 0.5-mM oxidized glutathione, and the enzyme solution. One unit of GR activity is defined as the amount of enzyme which oxidizes 1 µmole of NADPH per min.

#### Catalase activity

Catalase (CAT; EC 1.11.1.6) activity determination was carried out according to the method described by [23]. The method is based on monitoring the rate of decomposition of H_2_O_2_ at 30 °C. For CAT activity determination, suitable volume of crude enzyme was added to 1 mL of substrate mixture, which consisted of 0.02-M H_2_O_2_ in 50-mM phosphate buffer, pH 7.0. The decomposition of H_2_O_2_ was followed as a decline in absorbance at 240 nm for 1 min. One unit of activity was defined as the calculated consumption of 1 µmole of H_2_O_2_/min at 30 °C. The extinction coefficient of H_2_O_2_ was taken to be 43.6 M^−1^ cm^−1^.

#### Lactate dehydrogenase activity

Lactate dehydrogenase (LDH; 1.1.1.27) activity was measured spectrophotometrically at 340 nm by determining the rate of oxidation of NADH in the enzymatic conversion of pyruvate to lactate. The reaction was carried out in the potassium-pyruvate solution as described by [24].

### Lipid peroxidation

Level of lipid peroxidation was determined by measuring the formation malondialdehyde (MDA) using the method of [25]. The principle is based on the fact that MDA produced from the peroxidation of membrane fatty acid reacts with 2-thiobarbituric acid (TBA) to yield a pink-colored complex measured spectrophotometrically at 532 nm.

### Total glutathione content

The total glutathione content (GSH) was measured colorimetrically as shown by [26]. The cell homogenates were mixed with equal volume of 13% TCA. The precipitated proteins were removed by centrifugation at 2000 rpm for 10 min, and the supernatant was used for the assay of total GSH.

### Total protein concentration

Protein concentration was determined using bovine serum albumin as a standard [27].

### Genotoxicity assay

#### Comet assay (single-cell gel, SVG)

DNA fragmentation was measured by the alkaline comet assay, which is used to identify the individual DNA migration patterns [28, 29]. The basic steps of comet assay are as follows: A layer of 1% ordinary agarose was first applied to conventional microscope slides. The cell suspension (lysate) was then combined with 70 μL of 0.5% low melting point agarose and applied to the slides. All slides were immersed in chilled buffer solution (2.5-M NaCl, 100-mM EDTA, 10-mM Trizma base, NaOH was added to pH 10; 1% Triton X-100, 10% DMSO were freshly added). Slides were kept in lysing solution for up to 24 h at 4 °C in the dark. Slides were then placed in an electrophoresis tank and placed in in freshly prepared alkaline solution (300-mM NaOH and 1.0-mM EDTA, pH 13) for 20 min, and then electrophoresis was performed for 30 min at 25 V (0.79 V). Slides were washed three times in neutralizing buffer for 5 min (0.4 M Tris, pH 7.5). Slides were fixed in cold 100% ethanol, dried in air, and stained with ethidium bromide. Slides were examined by fluorescent microscope (Carl Zeiss Axioplan with epifluorescence using filter 15 BP546/12, FT580, and LP590). The extent of DNA migration for each sample was determined by simultaneous image capture and scoring of 50 cells at magnification 400 × using Komet 5 image analysis software developed by Kinetic Imaging, Ltd. (Liverpool, UK). Images of comets were taken using a closed-circuit digital camera (CCD). All samples were evaluated for the extent of DNA damage using the following parameters: tail length, tail DNA %, and tail moment.

### Statistical analysis

All data are reported as mean ± SD for *n* = 3–4 independent experiments. Data were statistically analyzed using one-way analysis of variance option in SAS 9.3. Significant differences among means were separated using Duncan’s test. The *p*-values of less than 0.05 were considered to be significant. For the cell viability assay, the statistical significance was assessed between samples and the negative control (vehicle cells) using the SPSS 11 independent *t*-test. A probit analysis for IC_50_ and IC_90_ was conducted using the SPSS 11 system.

## Results

The biochemical effects of four main treatments on MCF-7 cell lines were evaluated after 48 h of incubation at IC_25_ concentrations of *T. indica* extract (47.3 g/mL), TAM (0.8 µg/mL), and their co-treatments in the absence and presence of GSH.

### Evaluation of GSH levels and its antioxidant and detoxification enzyme activities of MCF-7 cells

The level of GSH and the catalytic activities of GST, GPx, and GR were determined in MCF-7 cell lines with four different treatments and untreated cells.

#### *T. indica* extract treatment

**Fig. 1:**
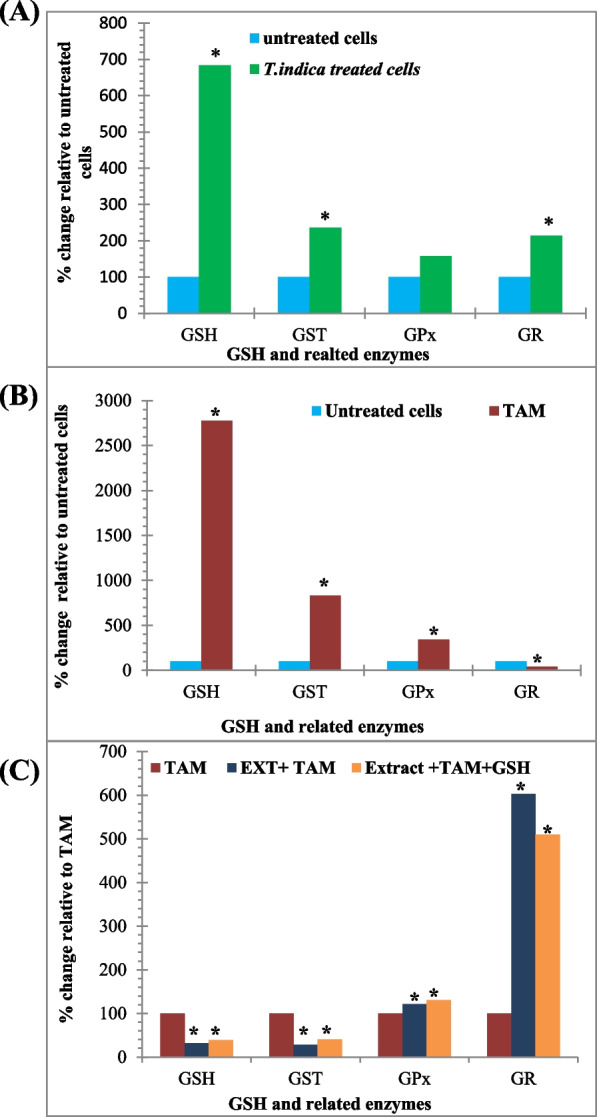
Effect of (**A**) *T. indica* extract, (**B**) TAM, and (**C**) co-treatment of *T. indica* extract + TAM and GSH on total GSH contents and its related enzymes; GST, GPx, and GR activities. Percentage (%) values calculated relative to untreated cells (**A**, **B**) and to TAM-treated cells (**C**). *Significant value *P* < 0.05

Treatment of MCF-7 cell lines with *T. indica* extract (47.3 g/mL) at IC_25_ concentration increased GSH level by more than 7-fold to 0.69 ± 0.034 µmol/mg protein compared to untreated cells, *P* < 0.05. Compared to untreated cells, the extract treatment increased the activity of GST, GPx, and GR to 1.1 unit/ mg protein (235%, 2.35-fold), 0.6 ± 0.26 unit/ mg protein (158%, 1.58-fold), and 0.204 ± 0.01 unit/ mg protein (214%, 2.15-fold), respectively (Table 1 and Fig. 1A).

**Table 1:** Level of GSH, and detoxification enzyme activities of MCF-7-treated cells

Treatment with IC_25_ concentration	GSH	GPx	GR	GST
**Untreated cells**	0.096 ± 0.012^d^	0.380 ± 0.03^c^	0.095 ± 0.03^b^	0.47 ± 0.06^d^
***T. indica*** **extract**	0.69 ± 0.034^c^	0.6 ± 0.26^c^	0.204 ± 0.01^a^	1.10 ± 0.02^c^
**TAM**	2.66 ± 0.024^a^	1.29 ± 0.40^b^	0.041 ± 0.005^c^	3.89 ± 0.43^a^
**Extract + TAM**	0.85 ± 0.21^bc^	1.56 ± 0.21^a^	0.245 ± 0.067^a^	1.11 ± 0.10^c^
**Extract + TAM + GSH**	1.03 ± 0.03^b^	1.68 ± 0.52^a^	0.207 ± 0.046^a^	1.57 ± 0.12^b^

The values represent the mean ± SD of three independent experiments. Values with different superscript letters within the same column indicate significant differences at *P* < 0.05. GSH level was expressed as µmol/mg protein, and enzyme activities of GST, GPx, and GR were expressed as µmole/min/mg protein. *GSH*, reduced glutathione; *GST*, glutathione S-transferase; *GPx*, glutathione peroxidase; *GR*, glutathione reductase

#### TAM treatment

The anticancer drug TAM, has a dramatic effect on GSH and its related enzymes of MCF-7 cells (*IC*_25_ = 0.8 g/mL), *P* < 0.05. GSH levels increased to 2.66 ± 0.024 μmol/mg protein, a 27.8-fold increase compared to untreated cells, and a 3.86-fold increase compared to *T. indica* treatment, *P* < 0.05. GST activity increased 8.33-fold to 3.89 ± 0.43 unit/mg protein as compared to untreated cells and 3.5-fold when compared to *T. indica* treatment, *P* < 0.05. The GPx activity showed a significant increase, with a 3.4-fold and 2.15-fold higher activity compared to untreated and *T. indica* treated cells, respectively (Table 1 and Fig. 1B)*.* This is the opposite of the effect of the increase observed with the previous parameters. The effect of TAM on GR activity was significantly reduced to 0.041 ± 0.005 units/mg protein with a 2.3-fold decrease compared to untreated cells and a 5-fold decrease compared to *T. indica* treatment, *P* < 0.05.

#### Co-treatment between *T. indica* extract and TAM

However, co-treatment of MCF-7 cells with TAM and *T. indica* extract reduced the effect on GSH levels and GST and GR activities to almost the same levels as *T. indica* extract single treatments. GPx activity was unaffected by such co-treatment. The antioxidant concentration of GSH (100 µM) increased GST and GPx activities to 1.57 ± 0.12 and 1.68 ± 0.52 unit/mg protein, respectively, while reducing GR activity to 0.207 ± 0.046 unit/mg protein (Table 1 and Fig. 1C).

### Evaluation of oxidative stress status of MCF-7 cells (CAT and LDH activities and MDA production as a biomarker of lipid peroxidation)

ROS generated in the cell during cellular metabolism can chemically react with cellular components such as nucleic acids, proteins, and lipids, generating oxidative changes and potentially damaging their biological activities. Cells, fortunately, have evolved many antioxidant defense mechanisms (as metabolites, vitamins, and enzymes) to neutralized or minimize the damaging effects of reactive species and/or their by-products. Any disruption in the balance of antioxidants and reactive species leads in a physiological condition known as “oxidative stress.” CAT is an important antioxidant enzyme that significantly reduces oxidative stress by decomposing cellular hydrogen peroxide to create water and oxygen.

Activities of enzymatic markers of oxidative stress and tissue damage conditions such as CAT and LDH as well as the amount of MDA production were determined as an indicator of lipid peroxidation status in MCF-7 cell line after 48 h of four different treatments and untreated cells.

#### *T. indica* extract treatment

**Fig. 2:**
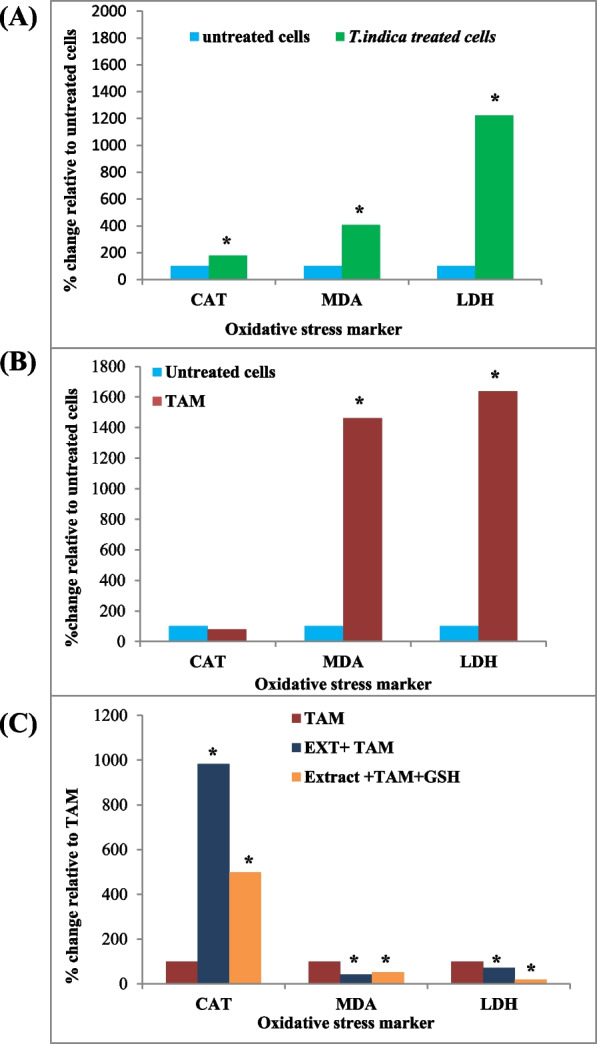
Effect of (**A**) *T. indica* extract, (**B**) TAM, and (**C**) co-treatment of *T. indica* extract + TAM, and GSH on the state of oxidative stress of MCF7 cells (CAT, LDH activities, and MDA production levels). Percentage (%) values calculated relative to untreated cells (**A**, **B**) and to TAM-treated cells (**C**). *Significant value *P* < 0.05

The activity of CAT in cells treated with *T. indica* extract was found to be slightly higher (2.72 ± 0.39 unit/mg protein) than in untreated cells (1.53 ± 0.13 unit/mg protein). LDH activity recorded a significant increase in activity in treated cells (0.147 ± 0.007 units/mg protein, 54-fold) compared to untreated MCF-7 cells, *P* < 0.05. In comparison to untreated cells (0.46 ± 0.029 μmol/mg protein), the level of MDA in cells treated with *T. indica* extract was significantly increased to 1.87 ± 0.18 μmol/mg protein (4.07-fold) (Table 2 and Fig. 2A).

**Table 2:** Oxidative stress status and viability of MCF-7 cells and CAT, LDH activities, and MDA production as a biomarker of lipid peroxidation

Treatment at IC_25_ concentration	CAT	LDH	MDA production
**Untreated cells**	1.53 ± 0.13^d^	0.012 ± 0.0004^d^	0.46 ± 0.029^e^
***T. indica*** **extract**	2.72 ± 0.39^c^	0.147 ± 0.007^b^	1.87 ± 0.18^d^
**TAM**	1.23 ± 0.09^d^	0.196 ± 0.018^a^	6.69 ± 0.64^a^
**Extract + TAM**	12.1 ± 0.06^a^	0.139 ± 0.008^b^	2.79 ± 0.28^c^
**Extract +TAM + GSH**	6.12 ± 0.85^b^	0.039 ± 0.002^c^	3.53 ± 0.35^b^

The values represent the mean ± SD of three independent experiments. Values with different superscript letters within the same column indicate significant differences at *P* < 0.05. CAT and LDH activities were expressed as µmole/min/mg protein, and level of MDA production was expressed as µmol/mg protein. *CAT*, catalase; *LDH*, lactate dehydrogenase; *MDA*, malondialdehyde production

#### TAM treatment

TAM treatment does not affect CAT activity (1.23 ± 0.09 unit/mg protein) as compared to untreated cells. TAM treatment dramatically increased MDA levels (6.69 ± 0.64 μmol/mg protein) compared to untreated cells (14.6-fold) and treated cells with the extract (3.6-fold). TAM treatment also significantly increased LDH to 0.196 ± 0.018 units/mg protein with a 72.3-fold increase in activity compared to untreated cells (Table 2 and Fig. 2B).

#### Co-treatment between *T. indica* extract and TAM

TAM and *T. indica* extract co-treatment significantly increased CAT activity to 12.1 ± 0.06 unit/mg protein: 9.8-fold higher than TAM treatment and 4.4-fold higher than extract treatment, *P* < 0.05. When GSH was added to the tamoxifen + *T. indica* extract treatment, it had the same enhanced effect on CAT activity as each single treatment and untreated cells, *P* < 0.05 (Table 2 and Fig. 2C). In contrast to the increased effect of TAM + *T. indica* observed with CAT activity, such combined treatment resulted in a significant decrease in LDH activity (0.139 ± 0.008 unit/mg protein) and levels of MDA production (2.79 ± 0.28 μmol/mg protein) compared with tamoxifen single treatment, *P* < 0.05. Addition of GSH to such a combined treatment decreased MDA production (3.53 ± 0.35 μmol/mg protein) compared to a single tamoxifen treatment; however, there was a significant decrease in LDH activity (0.039 ± 0.002 units/mg protein) with a similar value for untreated cells (Table 2 and Fig. 2C).

### Evaluation of genotoxicity and DNA damage of MCF-7 cells

#### *T. indica* extract treatment

**Fig. 3:**
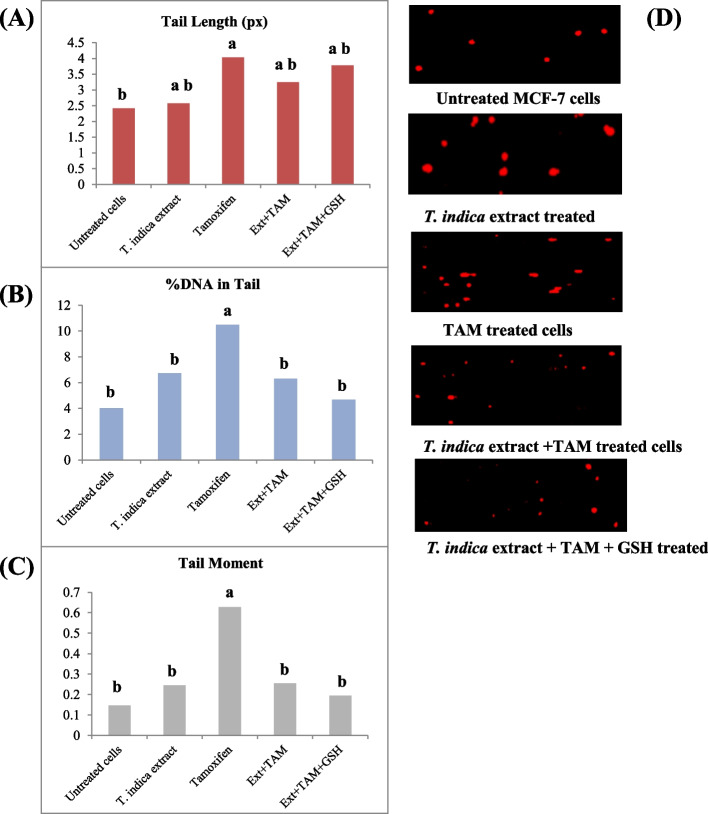
Effect of *T. indica* extract, TAM, and co-treatment of *T. indica* + TAM and GSH on DNA damage parameters. (**A**) Tail length (tail length is measured from the centre of the head to the end of the tail (µm), (**B**) tail DNA% (tail DNA % = 100* tail DNA intensity/cell DNA intensity), and (**C**) tail moment (tail moment (µm) = tail length × % of DNA in the tail) of MCF-7 cells. Column with different superscript letters indicates significant differences at level *P* < 0.05. (**D**) DNA damage detected by comet assay

DNA damage parameters (tail length, % tail DNA, tail moment) in untreated cells and MCF-7 cells treated with *T. indica* extract for 48 h were evaluated (Fig. 3). There was a slight increase in the examined DNA damage parameters (insignificant change, *P* ˃ 0.05) (Fig. 3A, B, and C).

#### TAM treatment

**Fig. 4:**
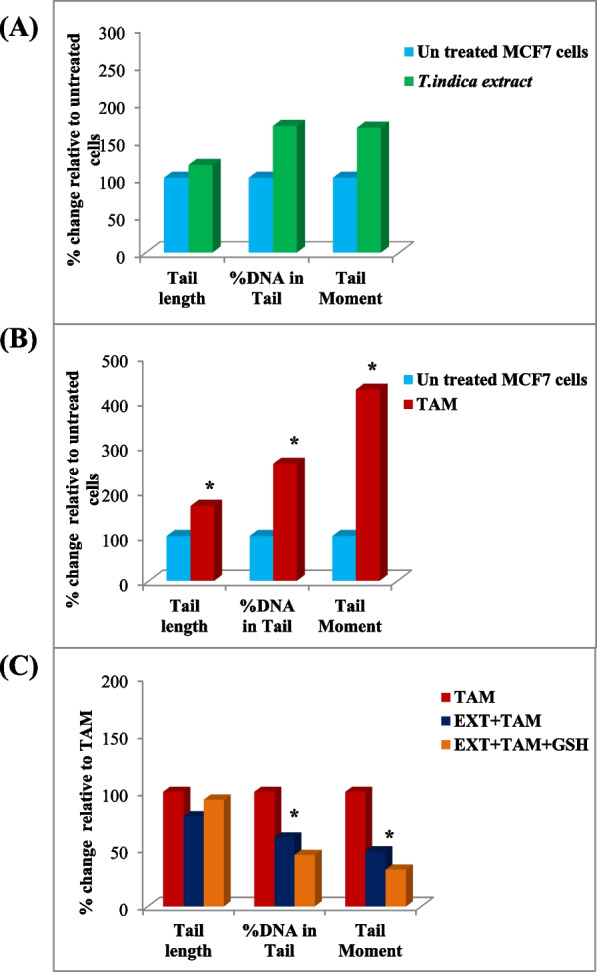
Effect of (**A**) *T. indica* extract. (**B**) TAM and (**C**) co-treatment of *T. indica* extract + TAM and GSH on DNA damage parameters: tail length (tail length is measured from the center of the head to the end of the tail (µm), tail DNA% (tail DNA % = 100* tail DNA intensity/cell DNA intensity), and tail moment (tail moment (µm) = tail length × % of DNA in the tail) of MCF-7 cells. Percentage (%) values calculated relative to untreated cells (**A**, **B**) and to TAM-treated cells (**C**). *Significant value *P* < 0.05

However, TAM treatment significantly increased the three DNA damage parameters when compared to untreated cells with 1.7-, 2.6-, and 4.26-fold increase in tail length, tail DNA%, and tail moment, respectively (Figs. 3 and 4).

#### Co-treatment between *T. indica* extract and TAM

The combined treatment of TAM and *T. indica* extract reduced the effect of TAM damage on the tested DNA damage factors. Significant decreases in % DNA in tail and tail moment values were observed by 60% and 48%, respectively, compared to TAM-treated cells, *P* < 0.05. Addition of the antioxidant GSH to such a combination treatment reduced % DNA and tail moment values by 45% and 32%, respectively, compared to tamoxifen-treated cells (Figs. 3B, C and 4C).

## Discussion

Cancer has been a major source of public concern as it has become the leading cause of morbidity and mortality worldwide. More than 60% of cancer cases are found in Africa, Asia, Central, and South America. According to the International Agency for Research on Cancer, there were around 1.1 million cancer cases and 717,000 cancer deaths in Africa in 2020 [30]. Prostate, lung, and colorectal cancers account for over half of all incidence cases in men, with prostate cancer alone accounting for 27% of diagnoses. Breast cancer, lung cancer, and colorectal cancer account for 51% of all new diagnoses, with breast cancer accounting for about one-third of all female diagnoses. As a result, an early biomarker for breast cancer diagnosis, prognosis, and a potential therapy target is necessary [31]. Cancer cells are not like normal cells in that they lack “intelligence” as a result of division and will proliferate uncontrollably and forced to exist in aerobic glycolysis settings. Tumor tissues can convert approximately ten times more glucose to lactate in a given time than normal tissues under aerobic conditions [32].

ROS are radical and non-radical chemical species generated by partial oxygen reduction that accumulates physiologically in parallel with cellular aerobic respiration. These radicals, if not controlled, may cause DNA damage and cell death. ROS can cause a variety of DNA damage, such as oxidized purines and pyrimidines, single-strand breaks, double-strand breaks, and basic sites [33]. Drug-derived reactive oxygen species (ROS), formed as a result of oxidative metabolism, are strongly associated with the inflammatory response. The body has defense mechanisms against ROS, such as certain enzymes (e.g., superoxide dismutase, SOD, CAT, and GPx, which generates biologically active metabolites), and thus plays an important role in the neutralization of ROS [34]. Oxidative stress is caused by an imbalance in the oxidant-antioxidant balance, which leads to an increase in the generation of free radicals, notably ROS, and, as a result, damage to biological components. As a result, oxidative stress has been linked to the pathogenesis of a variety of disorders, including cancer [7].

In the current study, levels of GSH and its related enzymes, as well as indicators of oxidative stress status and genotoxicity (DNA damage), were examined in cell line homogenates. *T. indica* extract increased GSH levels as well as GST, GPx, and GR activities in MCF-7 cell lines (Table 1). The same was true for oxidative stress state indicators, in which increased CAT and LDH activities correlated with increased level of MDA production (Table 2). However, this extract had a minor effect on the studied DNA damage parameters (Figs. 3 and 4).

Phenolic compounds have anticancer potential primarily because of their antioxidant activity; they are powerful radical scavengers, metal chelators, modulators of endogenous defense systems, inducers of GSH redox status, and regulators of numerous proteins and transcriptional factors [35]. The phytochemical composition of *T. indica* contains several classes of phenols, which provide its essential pharmacological properties and antioxidant activities. The ethanol extract of *T. indica* contains phenolics and flavonoids such as *p*-coumaric acid, protocatechuic acid, rutin, quercetin, and catechins [36]. In vitro, the antioxidant effect of *T. indica* extract and its ability to reduce lipid peroxidation were reported [17] study. According to [36], *T. indica* has high phenol contents and metal ion (Mn^+2^) levels, with an excellent dual effect of antioxidant and pro-oxidative activities, as well as cytotoxic effects on MCF-7 cancer cells. *T. indica* extract also increased the efficacy of the in vivo antioxidant resistance system as measured by SOD, GPx, and CAT activities, indicating that *T. indica* extract has therapeutic properties that may reduce the risk of many chronic diseases in humans [17].

GSH is an essential tri-peptide that works in the detoxification system through the GST catalytic reaction and in the antioxidant system through the GPx catalytic reaction. The cellular GSH pool is utilized by both enzymes, but after exposure to increased oxidative stress, GSH interacts with ROS and oxidizes, forming oxidized glutathione (GSSG), reducing the GSH/GSSG ratio that is decreased in many cancers [33, 37]. The sole enzyme that catalyzes the recovery of GSH from GSSG in a NADPH-dependent manner is GR [37]. This could explain the increased GR activity observed in the current results for MCF-7 cells treated with *T. indica* extract to meet elevation of GSH, thus increasing GPx activity.

Induction of ROS in cancer cells is conceived as a promising pharmacological approach to treat cancers. Because cancer cells have a higher basal ROS level than normal cells, proper dosing of ROS inducers may increase the ROS to a lethal level in cancer cells but a sublethal level in normal cells, hence selectively kill cancer cells. There are many anticancer agents, which kill cancer cells mainly or partly via induction of cellular ROS [38].

*T. indica* extracts (n-butanol and ethanol) were reported to be cytotoxic on MCF-7 cell lines, with 72% and 45.5% of dead cells, respectively, as well as their potential to operate as both an antioxidant and a pro-oxidant [18, 36]. The cytotoxicity of methanolic *T. indica* seed extract on two cancer cell lines (rhabdomyosarcoma and human lymphoma) was also indicated its anticancer efficacy. The presence of caffeic acid and other polyphenols in *T. indica* seeds can increase the antioxidant activity of treated cancer cells, protecting them from oxidation. It can also have an effect on cell viability, cell cycle, migration, phosphorylation, and gene expression [15]. Furthermore, consuming phytoestrogen-rich foods like *T. indica* has been proven to prevent the development of hormone-dependent breast cancer. Also, *T. indica* methanolic extract showed cytotoxicity against MCF-7 cell lines, as well as positively modulating progesterone production and negatively modulating estrogen levels in a time-dependent manner [39]. This is also true for the in vitro results using the methanol extract of *T. indica* seed coat which has a promising role in breast cancer prevention [39].

In this study, the increase in GSH and related enzymes (GST, GPx, and GR) is due to the ability of *T. indica* extract to act as a free radical producer (pro-oxidant activity), thus stimulating the antioxidant system. The increased level of lipid peroxidation (increased MDA levels) observed in the current study supports an imbalance between generation and removal of free radicals. This stimulation and imbalance within the cancer cells treated with *T. indica* extract could explain the higher activity of the damaged cell marker LDH, as well as the previously documented cytotoxicity as well as the unaffectedness of DNA damage parameters. These results indicate that the extract of *T. indica* can be used to develop new compounds against hormone-dependent breast cancer as it is possible that the effect of the plant extract on cancer cells only without normal cells.

Chemotherapy is based on antitumor drugs, which, by various mechanisms, interfere with the growth of cancer cells. However, chemotherapy shows many limitations as it is not limited to cancer cells but rather destroys healthy tissues resulting in their loss. In addition, cancer often develops resistance to treatment, resulting in treatment failure and disease recurrence. Chemotherapy drugs have significant limitations in cancer treatment, including drug solubility and instability, nonspecific drug delivery, and systemic toxicity. Chemotherapy drugs are classified as alkylating agents, anti-metabolic, immunomodulators, hormonal components, or antibiotics based on their mode of action. Endocrine therapy is used when patients’ cancer cells have positive hormone receptors. An estrogen receptor antagonist (e.g., TAM) is a class of drugs used in such therapy [2, 40].

In breast cancer, estrogen can bind to its receptor (ER), and this association provides a stimulus for the proliferation of breast cells, increasing the risk of mutations during DNA replication. 17-β-Estradiol (E2) binds with its receptors to specific regions of DNA, regulating the expression of several genes involved in critical processes such as cell cycle regulation, development, DNA replication, differentiation, and apoptosis [2]. TAM exerts antitumor efficacy independent of estrogen receptor (ER) expression through various mechanisms, such as those related to oxidative stress. TAM stimulates the generation of ROS and oxidative stress that can lead to damage to cellular structures and contribute to the adverse effects of TAM. Accumulation of TAM and its metabolites increases oxidative stress in breast cancer cells, leading to cell death. Cancer cells enhance TAM resistance by increasing levels of antioxidant enzymes and elevated levels of proteins that protect against ROS [16].

In this study, TAM treatment increased all redox balance parameters examined except for GR where a 43% decrease in its activity and a slight decrease in CAT activity were observed. TAM treatment also affects the three indicators of DNA damage with an increase in the value of the tail moment by more than fourfold. These results are in agreement with what has been previously reported in the literature and relate to the effect of anticancer drugs on the oxidative stress system and genotoxicity.

It was shown that GR activity declines dramatically with age and in several disorders linked with increased oxidative stress as compared to other enzymes in the GSH system. GR is necessary in healthy cells to protect them from oxidation. As a result, inhibiting or activating GR activity causes problems with the antioxidant system and several enzymatic reactions. The enzyme system containing GST and GPx utilizes the cellular GSH pool to induce detoxification or antioxidant responses by inactivating xenobiotics and oxidants under normal physiological conditions. In pathological situations, infected cells activate the glutathione system efficiently to survive; increased glutathione levels and dependent enzymes impair therapeutic efficiency. As a result, inhibiting GR enzymes is an essential therapeutic target in the fight against various cancers. Overexpression of the antiapoptotic protein Bcl-2 (B-cell lymphoma 2 (Bcl-2) homologous antagonist killer) raises the level of cellular and mitochondrial NADPH, which serves as a cofactor in the GSSG/GSH absorption process. The higher quantity of NADPH enhances mitochondrial GSH uptake, and the Bcl-2-GSH connection prevents apoptosis. Because of this mechanism, cancer cells become more resistant to chemotherapy and therefore enhances antioxidant capacity and resistance to oxidative stress [37]. This concept could explain the decrease and increase in GR activity reported in our results as a result of TAM treatment and TAM + extract co-treatments of MCF-7 cells vs. untreated cells.

As a consequence, combination therapy with polyphenols as an adjuvant in order to enhance the anticancer effect of commercial drugs is one attractive strategy to overcome the problem of resistance [41]. Combination techniques such as combining polyphenols with two or more polyphenols, combining polyphenols with anticancer drugs, combining polyphenols with vitamin supplements, or combining polyphenols with other efficacy have the potential in cancer treatment and drug resistance prevention. These techniques may help to slow growth of cancer cells, and in some situations, the combination compounds can work synergistically [42]. Combination therapy with several drugs is a common practice in cancer treatment. It is the best cancer reduction strategy in clinical chemotherapy. In fact, potential positive outcomes of synergy include the following: (1) increasing the efficacy of a therapeutic effect, (2) reducing the dose but increasing or maintaining the same efficacy to avoid toxicity, (3) reducing or slowing drug resistance development, and (4) providing synergy selectivity against target (or synergistic efficacy) versus toxicity (or antagonism) [43].

The current study examined the effects of co-treating *T. indica* extract with TAM in the absence or presence of GSH. GSH was added to the combination treatment between the extract and tamoxifen, believing that adding GSH could mitigate the side effects of this union, as GSH is available in the form of tablets to raise the efficiency of antitoxic systems. Co-treatment of *T. indica* with TAM reduced GSH level and GST activity, whereas this increased GR and CAT activity, accompanied by a decrease in LDH activity and MDA production). These findings of reduced oxidative stress status and MDA production supported the findings of the attenuating effect of the toxicity of the combined treatment (*T. indica* extract + TAM) previously reported in our work [18]. The same has been reported for the DNA damage agents examined; this combined treatment decreases TAM’s adverse effect.

CAT and GPx can both detoxify H_2_O_2_. CAT is found mostly in peroxisomes, whereas GPx is found primarily in the cytoplasm. This cellular distribution is definitely critical for H_2_O_2_ detoxification. Furthermore, the high affinity of GPx for H_2_O_2_ (km = 6 mM) compared to the low affinity of CAT for H_2_O_2_ (km up to 25 mM) suggests that GPx activates at low concentrations, whereas catalase, with low affinity, is activated at high concentrations. Thus, GPx and CAT alone are not enough to provide cellular defense; their cooperative interaction appears to be essential. Catalase’s susceptibility to H_2_O_2_ appears to be dependent on the amount of GPx activity. In the presence of H_2_O_2_, the upregulation of GPx keeps catalase activity high. The involvement of catalase in H_2_O_2_ clearance minimizes the depletion of intracellular GSH, which would otherwise limit the activity of GPx [44].

Cancer cells enhance TAM resistance through elevated levels of enzymes that protect against ROS. Increased levels of antioxidant proteins were observed in TAM-resistant MCF-7 cells when compared with nonresistant MCF-7 cells through Nrf2/antioxidant response element (ARE) activation. However, in response, breast cancer cells increased the expression of Nrf2 (nuclear factor (erythroid-2 related) factor 2), which activated ARE and increased transcription of genes related with anti-oxidation and multidrug resistance transporters, enhancing survival from tamoxifen-induced oxidative damage [16, 45].

To date, the development of the acquired resistance to cancer tends to be associated with increased cellular oxidative stress. Cancer cells that are drug resistant have a higher concentration of ROS and are more sensitive to changes in ROS levels. Compounds that can reduce the formation and accumulation of ROS have the potential to be effective in treating chemoresistance. As a result, the use of natural antioxidant molecules such as polyphenols to create the ROS barrier has become important. The antioxidant properties of polyphenols occur by stabilizing free radicals [2]. Polyphenols have the potential to influence various aspects of cancer drug resistance; including increasing drug uptake, decreasing drug metabolism by enzymes (e.g., cytochrome-c and GST), and decreasing drug efflux. Polyphenols also affect other chemoresistance targets in cancer cells, such as autophagy and apoptosis [9].

The current study found that GSH depletion was associated with reduced lipid peroxidation, and normal DNA appearance, implying that lipids play a significant role in resistance to H_2_O_2_-induced apoptotic cell death. This acquired resistance to oxidative stress could be due to intrinsic factors such as inability to initiate apoptotic pathways in cancer cells or activation of protective pathways such as antioxidant enzyme systems that eliminate ROS before they can act on the cells. We give evidence for this possibility in this study.

The current study differs from those of other studies interested in the same combination theory such as the results of [41] on the combination effect of some flavonoids with the anticancer drug cisplatin; the potency of a flavonoid named artocarpine has been suggested to enhance the anticancer activity of cisplatin on H460 and MCF-7 cell lines with morphological changes indicative of apoptosis. Treatment with oleuropein (a phenolic component found in olive oil) also decreased the development of human breast cancer cells BT-474, MCF-7, and T-47D, and the combined treatment oleuropein-tamoxifen inhibited the growth of the same cell lines in a synergistic manner. Natural substances such as hesperidin, nobiletin, tangeretin, and naringin, when combined with tamoxifen, have a synergistic effect on the MCF-7 cell line. Apoptosis, cell cycle regulation, and antiangiogenic effects are among the modes of action implicated [2]. Furthermore, co-treatment of antioxidants such as riboflavin and niacin with tamoxifen could improve its anticancer efficacy, as demonstrated in a Sprague–Dawley rat model of breast carcinogenesis where it restored lipid peroxide levels and enhanced antioxidant activity accompanied by antitumor activity. In contrast to what has been shown in tamoxifen-treated female Sprague–Dawley rats, tamoxifen-phospholipid compound can attenuate tamoxifen’s hepatotoxicity by reducing markers of toxicity such as lipid peroxidation or increasing the activity of antioxidant enzymes [16].

Antioxidants have the potential to reduce the efficacy of anticancer therapy. Cancer cells frequently accumulate more vitamin C than normal cells, suggesting that they may be better protected against the detrimental effects of ROS. The capacity of vitamin C to protect cancer cells against tamoxifen-induced lipid peroxidation in a breast cancer model in vitro supports the potential adverse effects of vitamin C supplementation during cancer treatment (MCF-7 cells). Moreover, co-treatment with lycopene (a carotenoid hydrocarbon antioxidant) increased the level of SOD, CAT, GPX, and GSH and reduced MDA activity as well. Besides, the combination of sodium butyrate and tamoxifen led to the upregulation of the CAT, SOD, and GPx genes in rat bone marrow cells. Therefore, this combination therapy could be associated with modulating the genotoxic effects of tamoxifen by reducing oxidative stress [16].

In this study, an increase in CAT and GR activity demonstrated the potential to increase rates of oxidative stress within MCF-7 cells co-treated with tamoxifen + *T. indica* extract, suggesting handling with high concentrations of free radicals. These increases in the activity of such antioxidant enzymes resulted of the combined treatment indicate that breast cancer cells are resistant to the drug, and that the defense system is trying to overcome the emerging oxidative stress state. All of these variants can cause abnormalities in the genotoxicity of cancer cells and apoptotic pathways, which explains the return of DNA moment to normal levels.

## Conclusions

Finally, the results of this study confirmed the cytotoxic and genotoxic effect of treatment with *T. indica* extract on MCF-7 cancer cell lines, which could be attributed to the dynamic interaction of GSH cycle and antioxidant enzymes to combat oxidative stress caused by this extract, which can be considered as a positive therapeutic effect. On the other hand, the negative response of cancer cells towards the supposed enhancement of tamoxifen efficacy when co-treated with *T. indica* confirms the ability of these cells to protect them by activating GSH and the antioxidant response against an increased oxidative stress state. As a result, combined therapy reversed tamoxifen’s genotoxicity and enhanced survival. Since the antagonism and recovery of the majority of the markers and enzymes evaluated in this study were proven, it is important to avoid taking any nutritional supplements or eating a certain type of food or drink during chemotherapy. But the main and definitive reason for this antagonistic effect is not clear whether it is limited to the state of oxidative stress or other causes. More research is needed to understand the mechanism by which these chemicals cause this impact.

## Data Availability

Not applicable.

## Abbreviations

CAT: Catalase
GSH: Reduced glutathione
GST: Glutathione transferase
GPx: Glutathione peroxidase
GR: Glutathione reductase
LDH: Lactate dehydrogenase activity
MCF-7: Human Caucasian breast cancer
TAM: Tamoxifen
MDR: Multidrug resistance
ROS: Reactive oxygen species
